# Editorial: New exploration for therapeutic tissue engineering grafts in the field of tissue regeneration

**DOI:** 10.3389/fphar.2023.1256907

**Published:** 2023-07-27

**Authors:** Tianhao Yu, Aijun Wang, Qiang Ao

**Affiliations:** ^1^ Liaoning Provincial Key Laboratory of Oral Diseases, The VIP Department, School and Hospital of Stomatology, China Medical University, Shenyang, Liaoning, China; ^2^ Department of Neurological Surgery, University of California Davis, Sacramento, CA, United States; ^3^ Department of Tissue Engineering, China Medical University, Shenyang, Liaoning, China; ^4^ NMPA Key Laboratory for Quality Research and Control of Tissue Regenerative Biomaterial, Institute of Regulatory Science for Medical Device, National Engineering Research Center for Biomaterials, Sichuan University, Chengdu, Sichuan, China

**Keywords:** tissue damage, tissue repair, tissue engineering, therapeutic grafts, regenerative medicine

Restoration following various types of tissue damage is still a great challenge in the clinic (Guan et al., 2022; Javed and Ao, 2022). Unsatisfactory functional recovery seriously affects the quality of life of patients and has puzzled both doctors and researchers worldwide (Zhu et al., 2021). The rise of tissue engineering in recent decades bring new prospects for addressing this concern. Tissue engineering attempts to repair, replace, or regenerate tissues and organs damaged by injury or disease through engineering and biological principles for therapeutic purposes (Zhang et al., 2016). In recent years, with the development of life sciences, medicine, material sciences, and engineering, therapeutic tissue engineering grafts have made rapid progress in several aspects of tissue repair (Yu et al., 2022). The continuing exploration of optimizing tissue engineering grafts in terms of biochemical and biophysical cues opens up more possibilities to impart them superior repair functional properties (Wang and Sakiyama-Elbert, 2019; Kihara et al., 2022). In addition, the incorporation of topological cues to mimic native tissue microstructure also plays a positive role in further enhancing the therapeutic effects of tissue engineering grafts (Sun et al., 2019). However, the development of therapeutic tissue engineering grafts remains in its infancy, and only a few have reached the phase of preclinical research or clinical therapy so far (Lin et al., 2021). The exploration of therapeutic tissue engineering grafts that can comply with the underlying mechanisms of tissue regeneration and fully exploit the inherent repair potential of the human body is a major current research direction. The objective of this Research Topic was to gather high-quality basic and applied research papers about the design, preparation, and characterization of innovative therapeutic tissue engineering grafts as well as their potential application in regenerative medicine. In addition, this Research Topic also focuses on the application of novel technologies derived from tissue engineering for disease treatment as well as drug delivery, which have also been greatly advanced recently.

This Research Topic begins with an article by Lin et al., entitled “*Upper-critical-solution-temperature polymer modified gold nanorods for laser controlled drug release and enhanced anti-tumour therapy*.” The development of Photothermal therapy (PTT) system with a high anti-tumour effect is a feasible research direction. This study proposed a new type of gold nanorods (AuNRs)-doxorubicin (DOX)/mPEG_10K_-peptide/P (AAm-co-AN) (APP-DOX) nano drug delivery system. AuNRs is used as high-efficiency photothermal agent. APP-DOX has a suitable size and can be targeted to accumulate in tumour tissues through circulation in the body. The abundant matrix metalloproteinase 2 (MMP-2) in the tumour environment intercepts and cuts off the short peptide chain structure grafted on APP-DOX. At the same time, the removal of the PEG segment leads to an increase in the hydrophobic properties of the nanoparticles, causing them to stay and aggregate further at the tumour site. When irradiated by 808 nm near-infrared laser, APP-DOX achieved a gradual heating process. High temperature can effectively ablate tumours and enable Upper-Critical-Solution-Temperature polymer to achieve phase transition, resulting in the release of anti-cancer drugs loaded in the polymer layer DOX, effectively killing cancer cells. Animal experiments verify the possibility of the nano drug-carrying system and good tumour treatment effect. Moreover, compared with free DOX, the nano drug delivery system has lower biological toxicity and does not cause obvious harmful effects on normal organs and tissues.

Another noteworthy contribution to the Research Topic is a research article by Song et al., titled “*Engineered multi-functional, pro-angiogenic collagen-based scaffolds loaded with endothelial cells promote large deep burn wound healing*.” Large deep burn wounds are difficult to achieve adequate vascularization, which may delay the construction of wound beds, resulting in insensible fluid and heat losses, increased metabolic demand, increased risk of infection, and the formation of hypertrophic scar. To address this challenge, this study devised a multi-functional collagen-based Integra scaffold modified by integrin αvβ3 ligand LXW7- dermatan sulfate-collagen binding peptide SILY and loaded with endothelial cells (ECs). Its repair capacity was assessed using a large deep burn wound model in mice. The results demonstrated that the engineered scaffold not only facilitates the survival and proliferation of exogenously seeded ECs, but also accelerates the recruitment of endogenous ECs and angiogenesis, which contributes to establishing a superior wound base and promoting wound healing. The novel scaffold represents a potentially effective treatment for deep burn wounds with large areas. Not only that, the scaffold offers possibilities for reducing the requirement for autografting and its accompanying morbidity in patients with limited harvestable skin.

Mao et al. published an important research article titled “*Nerve ECM and PLA-PCL based electrospun bilayer nerve conduit for nerve regeneration*.” This study designed a novel electrospun bilayer-structured nerve conduit (BNC) with the outer layer of poly (L-lactic acid-co-ε-caprolactone) (PLA-PCL) and the inner layer of ECM for nerve regeneration. The composition, structure, mechanical strength, and biosafety of BNC were characterized. Then, BNC was used to bridge 10-mm rat sciatic nerve defect, and nerve functional recovery was assessed by walking track, electrophysiology, and histomorphology analyses. The results demonstrated that BNC has a network of nanofibers and retains some bioactive molecules, including collagen I, collagen IV, laminin, fibronectin, glycosaminoglycans, nerve growth factor, and brain-derived neurotrophic factor. Biomechanical analysis showed that PLA-PCL improved the mechanical properties of BNC compared with single ECM conduit (ENC). The functional evaluation of animal experiments indicated that BNC is more effective in nerve regeneration than PLA-PCL conduit or ENC. In conclusion, BNC not only retains the good biocompatibility and bioactivity of ECM, but also obtains the appropriate mechanical strength from PLA-PCL, which has great potential for clinical repair of nerve defects.

Finally, a research article by Adamiak-Giera et al., entitled “*Evaluation of the in vitro permeation parameters of topical ketoprofen and lidocaine hydrochloride from transdermal Pentravan products through human skin*” provides valuable insights into pain pharmacotherapy. It is always difficult to balance the effectiveness and safety of medication in the treatment of pain, motivating this study to explore the optimal route of drug delivery. The transdermal route has significant advantages over the conventional oral route, which may be an effective approach to prevent or minimize the potential side effects related to oral drug administration. Pentravan is a transdermal liposomal cream base, which can enclose molecules of the bioactive components and promote their transdermal delivery, providing a potentially optimal route for the use of topical drugs with appropriate composition and concentration. This study assessed the *in vitro* permeation of KET and LH through the Pentravan transdermal vehicle. The results showed that the Pentravan is a superior vehicle with transdermal properties, which can deliver a higher drug dose than other commercial products, and ensure rapid permeation of analgesic and anti-inflammatory drugs. The combination of Pentravan with certain drugs is a highly promising alternative to enteral drugs, especially for patients with multiple diseases and polypharmacy.

Altogether, the papers included in this Research Topic introduce and discuss the new strategies and application prospects of therapeutic tissue engineering grafts and their derived technologies. Although there is still a long way to transfer from bench to bedside, emerging studies offer creative pathways for the further development of tissue engineering. Broad interdisciplinary research and revolutionary methodological advancements are expected to pave the way for clinical translation.
